# 3D-printed low-cost choke corrugated Gaussian profile horn antenna for Ka-band

**DOI:** 10.1038/s41598-023-50174-5

**Published:** 2023-12-27

**Authors:** Yair D. Zárate, Francisco Torres, Mauricio Rodriguez, Francisco Pizarro

**Affiliations:** Escuela de Ingeniería Eléctrica, Pontificia Universidad Católica de Valparaíso, 2340000 Valparaiso, Chile

**Keywords:** Engineering, Electrical and electronic engineering

## Abstract

In this work, a fully 3D-printed choke corrugated Gaussian profile horn antenna (GPHA) using high-conductive filaments and a low-cost modular 3D-printing technique is implemented. The choke corrugated GPHA operates in the Ka-band, with a central frequency of 28 GHz. Although the antenna can be printed in one piece as its dimensions are within the printing limits, four pieces compose the three sections of the final 3D-printed antenna. The numerical simulations and measurements of the antenna show a good agreement, validating the possibility of cost-effective modular fabrication of this complex type of antennas.

## Introduction

In recent years, 3D-printing has been largely used in different applications, including electronics and electromagnetic devices^1^. The advances in this technology, such as the cost reduction of high-precision 3D printers plus the availability of low-loss dielectric filaments and conductive filaments, have allowed to implement prototypes or devices that were either to expensive or even impossible to implement. In the case of high-frequency electromagnetic devices we can find studies and implementations for dielectric resonator antennas^2^, lenses^3^, polarizers^4^, amongst other topologies. In addition, the progress of telecommunication technologies and its applications produce an ongoing demand for constant updates and advancements in the topologies and essential materials for high-frequency telecommunications in both terrestrial and satellite applications^5^. This pursuit of improvement does not only imply the upgrade or replacing of existing devices and topologies but also encompasses the exploration of materials and structures that minimize the cost, with simpler and lighter compact designs that are compatible with modern fabrication techniques^6–9^.

One basic topology that has had a direct benefit from additive manufacturing are horn antennas^10^. Through the years many designs have been proposed for horn type antennas, and they were manly focus on optimizing the antenna adaptation to the propagating modes of several waveguide geometries. This was accomplished by adapting their horn shape aperture to provide a gradual transition structure to match the impedance of a waveguide to the impedance of free space, enabling the confined waves from the waveguide to radiate efficiently into open air. The continued development of these aperture antennas considered the introduction of corrugations over the internal walls of the structures^11,12^. This brought an improvement not only in their capacity as mode transformer, but also enabled the reduction in size of these antennas making them a reliable first choice for applications in terrestrial and satellite telecommunication due to their lighter, more compact, and efficient designs^13–15^. Newer topologies of corrugated horn antennas explore the integration of mode converter within the antenna profile. Those structures feature, as a result, smoothly connected sections with distinct profile, producing an unified structure, in spite of these sections having different corrugation pattern and/or orientation (e. g. horizontal or transversal to the propagation axis) within the antenna^15–17^. These novel geometries have been developed, theoretically and empirically, to maximize antennas outcome, capable of meeting the highest demands in directivity, gain, and cross-polarization, with broad-and narrow-band of great performances, while reducing further the antennas dimensions for target working frequency^6,13^. Nevertheless, when implementing for complex structures, costs or weight can be limiting for some applications, and that is where 3D printing can have an impact into the cost-effectiveness of the the device.

One interesting horn antenna topology that can have a benefit from 3D-printing is the Gaussian Profile Horn Antenna (GPHA). This antenna, specially when operating in higher frequency bands, the fabrication precision are pushed to the limits due to the reduction of the wavelength in use^14,17,18^. In the literature, 3D-printed corrugated horns have been proposed using different techniques, such as laser sintering, fuse filament fabrication (FFF) with electrodeless deposition, stereolitography, conductive aerosol coating and others metallic 3D-printing techniques^19–24^, all techniques that can involve higher costs in their implementation due to the nature of its manufacturing, or post processing. Consequently, it becomes imperative to employ manufacturing techniques that are not only cost efficient but also modular in nature^25^, ensuring cost-effective solutions regarding the target application. Therefore, modern corrugated horn antennas are promising compact and cost-efficient topologies that address the performance required by future telecommunications technologies.

In this manuscript, a choke corrugated Gaussian profile horn antenna is designed and manufactured using low-cost 3D printing, in order to produce a cost-effective prototype working at Ka-band.

## Antenna design and simulation results

The antenna design corresponds to a choke corrugated Gaussian profile horn antenna, working at the Ka-band with central frequency $f_c=28$ GHz. The antenna, the choke transformer and the Gaussian-shaped horn are based on the work proposed in^26^ and^27^. The antenna design considers a choke mode transformer of conical shape that transforms circular mode, $TE_{11}$, into the hybrid $HE_{11}$ mode propagating inside the corrugated Gaussian-shaped horn^26^, and is subsequently transformed into the Gaussian beam by the aperture of the horn antenna. The walls of the choke section have horizontal corrugations, aligned to the axis of propagation. The horn aperture section of the antenna has a Gaussian profile, which can be described as a gradually expanding aperture with a smooth curved profile of hyperbolic shape, which interior is corrugated in the vertical direction (transverse to the propagation axis).

**Figure 1:**
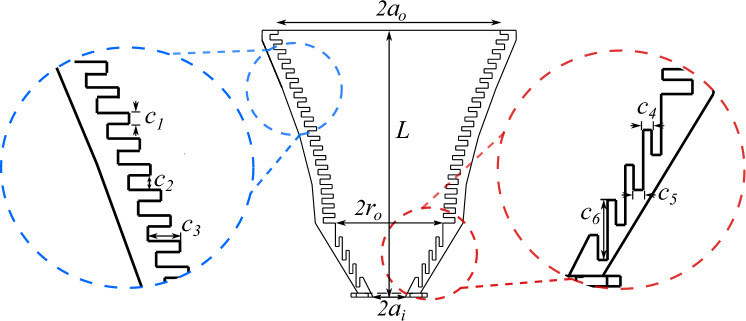
Antenna Diagram. The conical choke section is found at the bottom of the image, and its corrugations are horizontal to the axis of propagation. The Gaussian aperture, featuring vertical corrugations, connects with the choke transformer forming a transition of radius $r_0$. The values of the geometrical parameters of the antenna design are found in Table 1.

The corrugations that are implemented perpendicular to the wall aim to reduce the longitudinal currents on the boundaries, forcing nulls of the electric field at the edge of the opening, improving the antenna efficiency while reducing antenna dimensions. The purpose of the vertical corrugated surface of the Gaussian profile aperture is to provide efficient propagation of hybrid mode within the horn and minimizing the side lobes. The sketch of the proposed antenna is shown in Fig. 1.

### Geometrical parameters of the antenna

The proposed choke corrugated GPHA is composed of two sections: the choke transformer and the corrugated Gaussian horn aperture. The geometrical parameters of the antenna design have been optimized to maximize the conversion of mode generated at the choke input, into the Gaussian beam radiated by the horn antenna. The antenna is also designed to operate in the Ka-band, with a central working frequency of $f_c=28$ GHz. The corrugation period of the antenna is set to $2.4\simeq \lambda _c/4$, with $\lambda _c\simeq 10.7$ mm, the output diameter of $2\cdot a_o\simeq 5.2\lambda _c$ and total length of the antenna is $L\simeq 6.3\lambda _c$. The Gaussian geometry of the GPHA is determined by the following profile equation^26,28^

1 $$\begin{aligned} r(z)=r_0\sqrt{1+\left( \frac{\lambda _c\cdot z}{\pi \cdot \alpha \cdot r_0^2}\right) ^2} \end{aligned}$$

where $\alpha$ is a parameter that controls the slope of the Gaussian-shaped aperture, in this case, it has been set to $\alpha =0.5$. The radius of connection $r_0$ denotes the transition point between the choke section and the Gaussian profile, and its value is set to $r_0=27$ mm. The antennas choke section profile is determined by a line with a slope of 25$^\circ$, which starts in $a_i$ at the antenna input radius, where the feed waveguide is connected, and ends in $r_o$, where it connects with the antenna Gaussian aperture and the corrugations orientation transit from horizontal to vertical.

Therefore, this horn antenna has a reduced size when compared to other similar horns, with the disadvantage that the corrugations can imply a cost for its cost-efficient implementation when using traditional manufacturing techniques. The sketch of the choke corrugated GPHA geometry is shown in Fig. 1, and the value of the corresponding geometrical parameters. To notice that some values, such as the horizontal corrugation width, have been enlarged to fit the 3D printer limitations.

**Table 1:** Geometrical parameters of the optimized antenna design operating in the Ka-band.

Geometrical parameter	Label	Value (in mm)
Length	*L*	67
Input radius	$a_i$	4
Output radius	$a_o$	28
Connection radius	$r_0$	27
Vertical corrugation width	$C_1$	1]
Vertical sloth width	$C_2$	2.4
Vertical corrugation depth	$C_3$	3
Horizontal corrugation width	$C_4$	0.8
Horizontal slot width	$C_5$	1.7
Horizontal corrugation depth	$C_6$	2.4

### Simulation results

**Figure 2:**
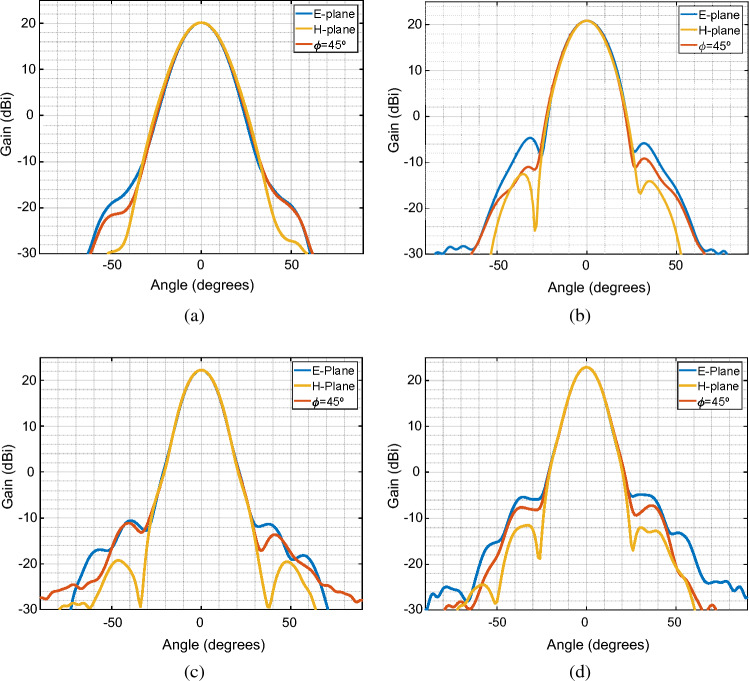
Simulated gain radiation pattern at four different frequencies and on three cut planes, namely E-plane, H-plane, and diagonal or $\phi =45^\circ$ (**a**) 28 GHz. (**b**) 30 GHz. (**c**) 32 GHz. (**d**) 34 GHz.

All simulations are performed using the full-wave software Ansys-HFSS. For simplicity, all conductive materials are set to perfect electric conductors (PEC). Figure 2 shows the gain radiation pattern for different frequencies over the band of interest, on three different cut planes of the antenna: E-plane, H-plane, and a diagonal or $\phi =45^\circ$ plane. We can see from the results that the antenna has a maximum gain of around 20 dBi at 28 GHz, while it keeps its symmetry on all three assessed planes. As expected, this geometry has very low sidelobe levels, which are below −20 dB in all the assessed frequencies.

## Antenna manufacture using 3D-printing

The antenna was fabricated using using modular 3D printing technique and the conductive filament Electrifi from Multi3D^29^. The 3D printer used is an Ocular3D EIE-custom with an enlarged CNC-machined nozzle which prevents damage caused by clogging of the conductive filament. The characteristics of the 3D-printer are shown in Table 2. Notwithstanding, it is possible to produce the full antenna in one piece because its details and dimensions are within the printing volume limits, this has been divided into four pieces forming the upper section of the aperture antenna. This is done to minimize errors and cost, as the printing speed and flow must be checked during the printer, and a larger printer process may cause a defective part. This division assures that only one section can be reprinted and not not the complete antenna. Finally, the pieces are joined by regular cyanoacrylate-based adhesives.

One final characteristic of this antenna is that in practice it will be fed by a standard WR-28 rectangular waveguide. In order to reduce reflections, a standard rectangular to circular waveguide transition section is added, also 3D-printed. Then, the antenna is composed by three sections: the transition, the conical choke transition section that features corrugations horizontal to the axis of propagation, and the Gaussian aperture section of the antenna. The antenna then is printed using the parameters shown in Table 3. To notice that the temperature and printing speed were set in order to avoid as much as possible any clogging on the hot-end when printing the larger sections of the antenna, and to have a good compromise in terms of the resulting surface roughness of the inner walls of the antenna. Finally, the infill percentage and pattern were set in order to assure mechanical stability of the corrugations of the antenna.

**Figure 3:**
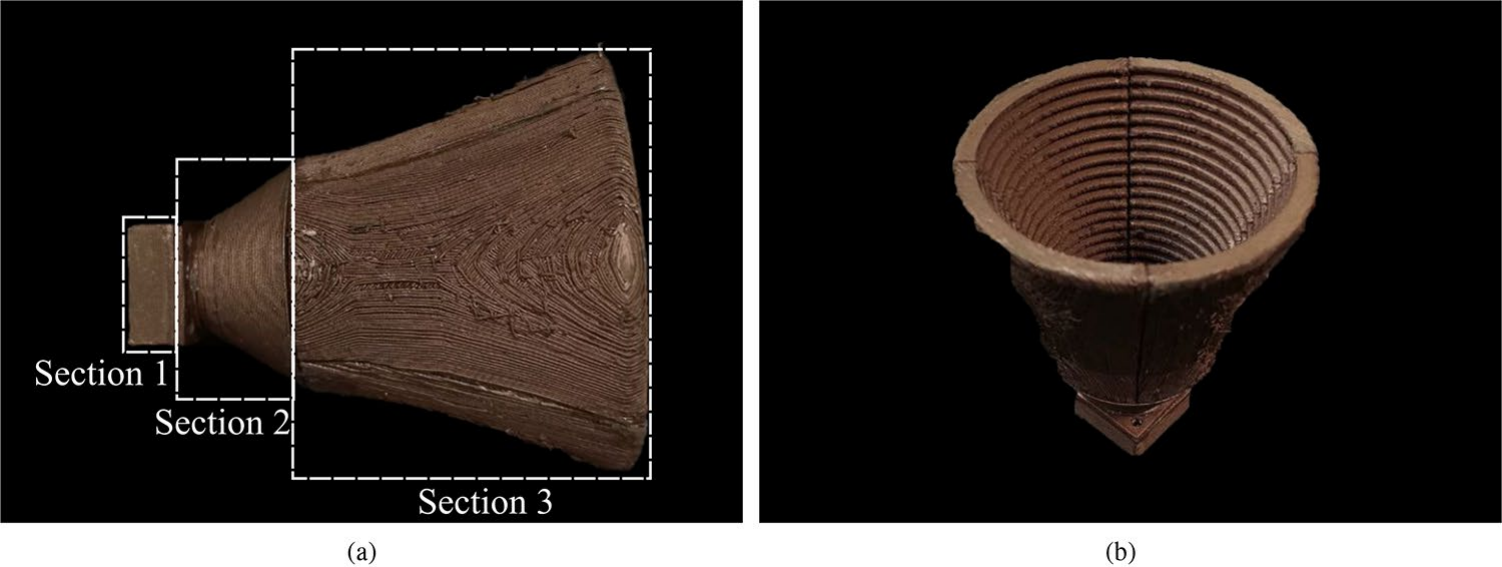
3D-printed choke corrugated GPHA. (**a**) Lateral view of the antenna configuration is composed of section 1; the rectangular to circular waveguide transition, section 2; the choke transformer, and section 3; the aperture of Gaussian profile. (**b**) Perspective view of the 3D-printed choke corrugated GHPA.

The lateral and perspective view of the joined 3D printed choke corrugated GPHA is shown in Fig. 3. We can see that the inner walls and corrugation are well printed, while all the imperfections remains on the outer walls, which has no effect over the performance of the antenna. Finally, the antenna overall weight is around 37 grams.

**Table 2:** Custom 3D printer technical specifications.

Printer parameter	Value
Maximum printing volume	120$\times$120$\times$190 mm$^3$
Axis resolution	100 $\upmu$m in all axis *(xyz)*
Nozzle diameter	0.4 mm
Filament diameter	1.75 mm
Hot-end T$^{\circ }$ range	120 to 260 $^{\circ }$C
Platform maximum T$^{\circ }$	100 $^{\circ }$C
Max. print speed	60 mm/s

**Table 3:** Printer configuration and parameters set based on the conducting filament requirements for correct deposition.

Printer configuration	Value
Layer height	0.2 mm
Infill percentage	100%
Fill pattern	Concentric
Printing temperature	$160\,^\circ$C
Printing speed	20 mm/s
Cooling fan	$100\%$

**Figure 4:**
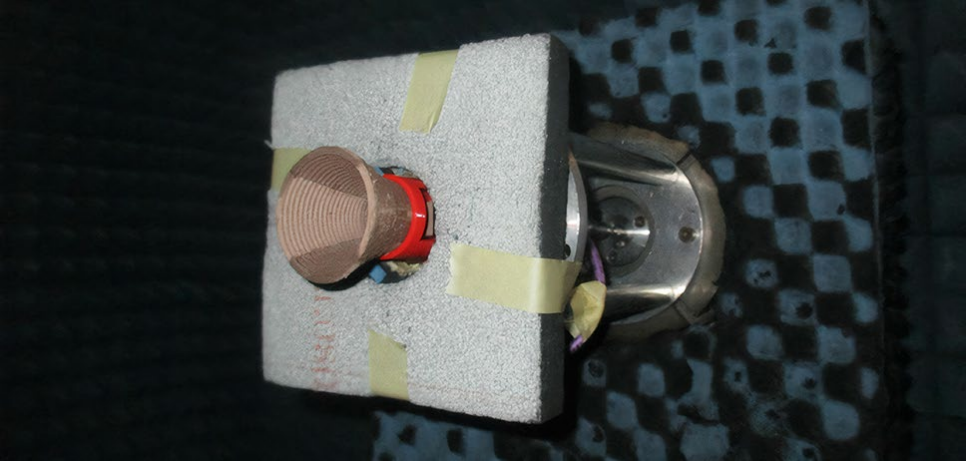
3D-printed antenna measurement in anechoic chamber.

## Measurement results

**Figure 5:**
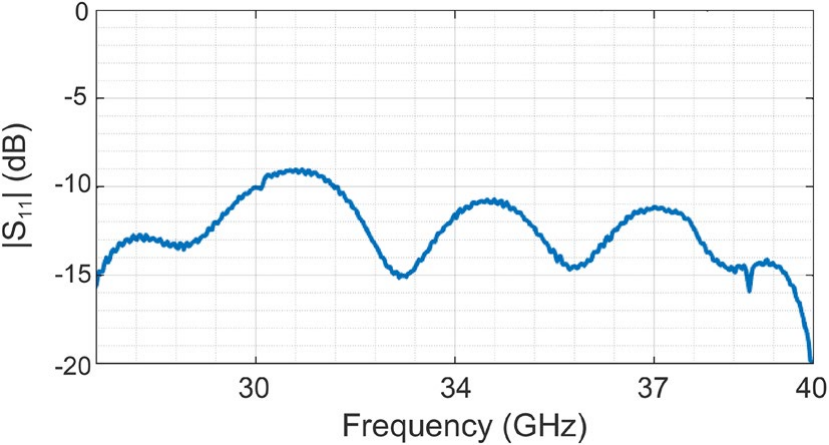
Measured reflection coefficient $|S_{11}|$ as function of the frequency.

**Figure 6:**
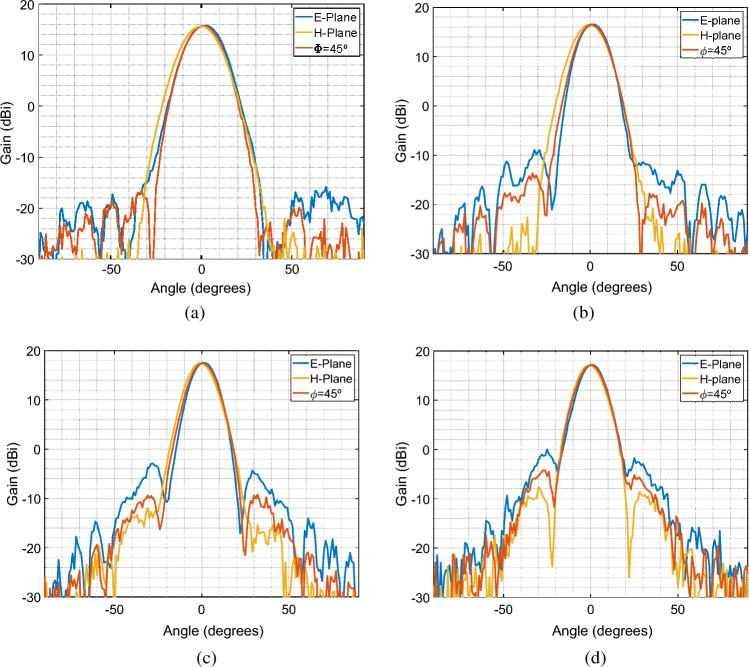
Measured gain radiation patter at three cut planes (E-plane, H-plane, and $\phi =45^\circ$) for different frequencies in the Ka-band. (**a**) 28 GHz. (**b**) 30 GHz. (**c**) 32 GHz (**d**) 34 GHz.

The antenna gain radiation pattern was measured in an anechoic chamber, as shown in Fig. 4, the measured reflection coefficient and the resulting radiation patterns are shown in Figs. 5 and 6 respectively. We can see that for the different frequencies in the Ka-band, the radiation pattern remains symmetrical for the three assessed cut planes (E-plane, H-plane, and $\phi =45^\circ$ plane). Therefore the 3D-printed choke corrugated GPHA feature a performance similar to that obtained with full-wave numerical simulations in terms of symmetry, half-power beamwidth, and low sidelobe levels (below 15 dB in all the assessed frequencies). This confirms that in terms of the dimensions of the antenna, any discrepancy that due to tolerance manufacture has not a large influence over the radiated field of the antenna.

Another important parameter to evaluate is the reflection coefficient, as it can be sensitive to dimensional changes that can create a mismatch on the antenna. In Fig. 5 is shown the measured reflection coefficient of the printed antenna. We can see that it is almost matched on over the whole band of operation, but a slight mismatch is introduced near 28 GHz, which can be explain due to dimensional tolerances or differences in the input section of the antenna. Nevertheles, this mismatch do not affect largely in the gain drop experience by the manufactured antenna.

**Figure 7:**
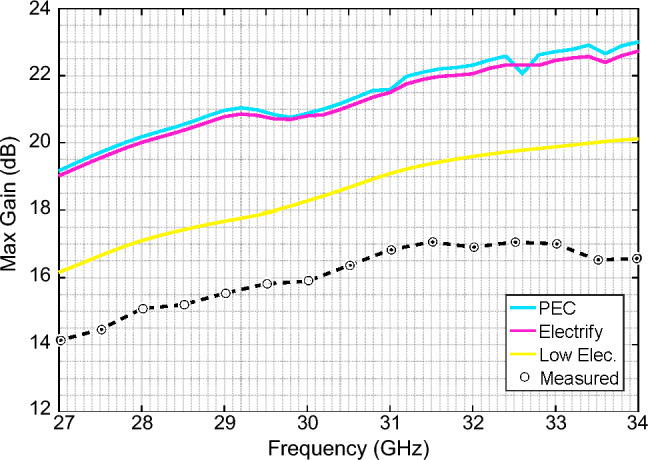
Measured and simulated maximum gain as a function of the frequency for different conductivity values.

The main difference between simulation and measurements are found on the overall maximum gain of the manufactured antenna. This gain is considerably lower when compared with the antenna simulated with PEC, which is expected due to the lower conductivity of the material. In order to evaluate those losses, in Fig. 7 shows the simulated maximum gain of the antenna as a function of the frequency, for three different conductivities, and compared with the measured results. The evaluated conductivities are PEC, the nominal filament conductivity estimated at $1.66\times 10^4$ S/m, and a lower conductivity value set to 750 S/m, namely Low Elec., which was observed at lower frequencies in previous studies^30^. From measurements, we can see a difference of around 6 dB when compared to the PEC case, and around 2 dB when compared to lower conductivity case. These differences can be explained by the following factors. First, The electrical conductivity of the printed structure strongly depends on the infill setup, printing pattern, and printing direction horizontal or vertical, this affect directly the fringes and vertical structures part of antenna design^29^. Second, surface roughness can be an important parameter when implementing antennas in higher frequency bands which introduces additional losses^31^. Additive manufacturing using fused filament fabrication has a surface roughness that its dependant mainly on the nozzle diameter and layer height. Previous studies have shown for simple 3D-printed waveguides printed with the same filament can have transmission losses as high as 0.35 dB/mm^32^. For this particular case, previous works done using this filament shows that the losses due to surface roughness can be considerably reduced using chemical post-processing, i.e., using chloroform based smoothing treatment, that has been proved to reduce drastically the surface roughness in waveguides implemented with this same filament.^32^.

## Conclusions

In this work, a corrugated choke Gaussian horn antenna was designed and implemented using low-cost 3D-printing and off-the-shelf conductive filaments, operating in the Ka-Band. The performance of the antenna in terms of its radiation pattern has a good agreement with what was expected from simulations. The overall gain reduction can be explained due to the lower conductivity value of the printed filament, as well as the surface roughness inherited from this low-cost manufacturing process. Nevertheless, the low cost of this antenna, which can be very difficult to implement using traditional manufacturing techniques, as well as the low weight of the resulting prototype (around 37 grams) makes this implementation a cost-effective alternative for antennas with symmetrical radiation patterns to be used for example, in unmanned vehicles applications.

## Data Availability

The datasets used and/or analyzed during the current study are available from the corresponding author upon reasonable request.
